# Supplementary material for: Intradermal but not intramuscular modified vaccinia Ankara immunizations protect against intravaginal tier2 simian-human immunodeficiency virus challenges in female macaques
Source: Nat Commun. 2023 Aug 8;14:4789. doi: 10.1038/s41467-023-40430-7 (PMC10409804; doi:10.1038/s41467-023-40430-7)
Supplement: Supplementary file 1 — Supplementary Information [file 41467_2023_40430_MOESM1_ESM.pdf]

Intradermal but not intramuscular MVA immunizations protect against intravaginal tier2 SHIV challenges in female macaques

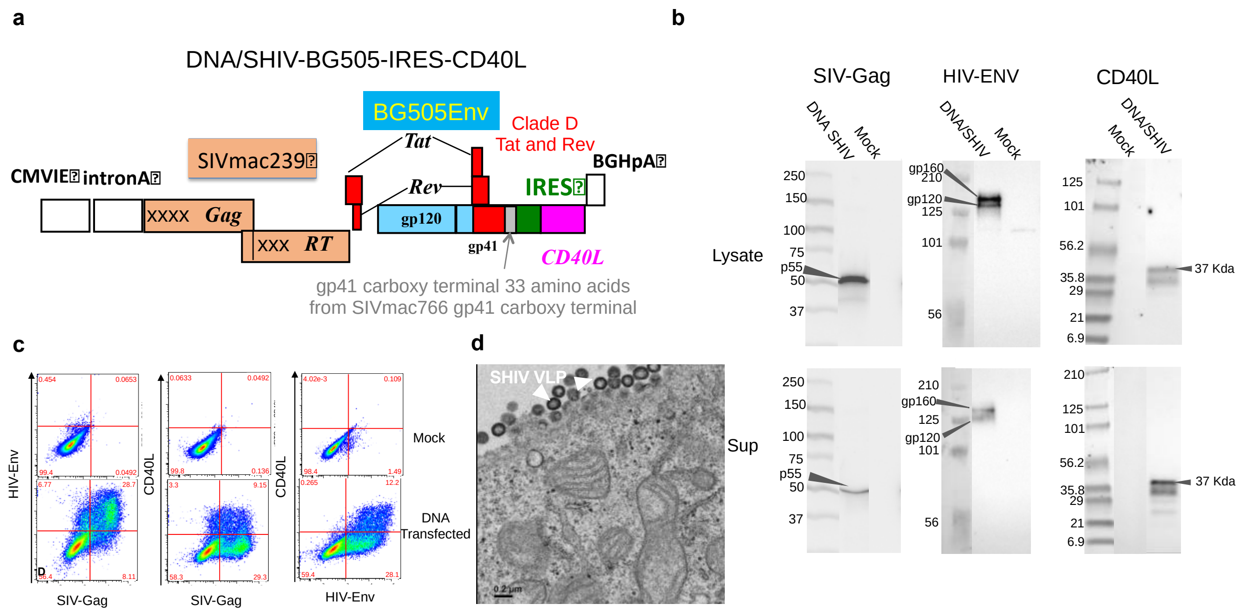

SFig. 1: DNA characterization

a) Schematic of SHIV BG505 DNA. b) Western blot confirmation of SIV-Gag, HIV-Env and rhesus CD40L protein expression in DNA transfected HEK-293T cell lysate and supernatant (Sup). The blots used to detect CD40L in the lysate and sup were run simultaneously on different gels. c) Flow cytometry confirmation of SIV-Gag, HIV-Env and CD40L surface co-expression on DNA transfected 293T cells. d) Electron microscopy images of SHIV BG505 VLPs budding from DNA transfected 293T cells.

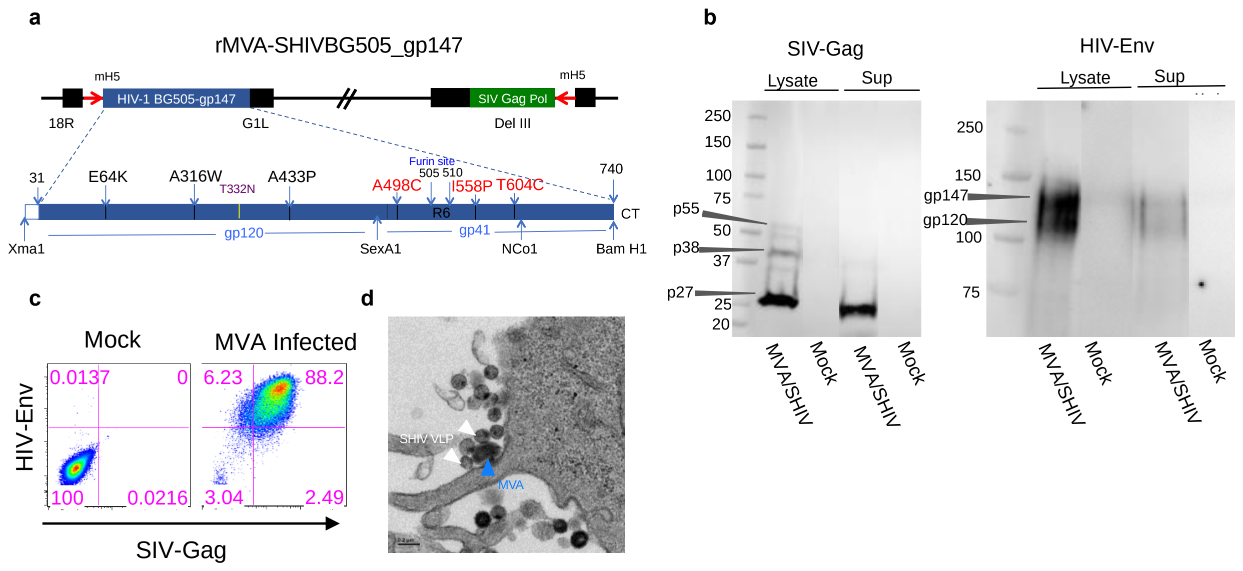

SFig. 2: MVA characterization

a) Schematic of SHIV BG505 MVA construct. b) Western blot confirmation of BG505 SHIV MVA construct expressing SIV-Gag and BG505-HIV Env proteins using anti SIV Gag antibody, 2F12 and anti BG505 rhesus serum respectively c) Flow-cytometry confirmation of SIV-Gag (stained with 2F12 antibody) and HIV-Env (stained with PGT121 antibody) surface co-expression on MVA infected DF1 cells. d) Electron microscopy images of SHIV BG505 VLPs and MVA viral particles budding from MVA infected DF1 cells.

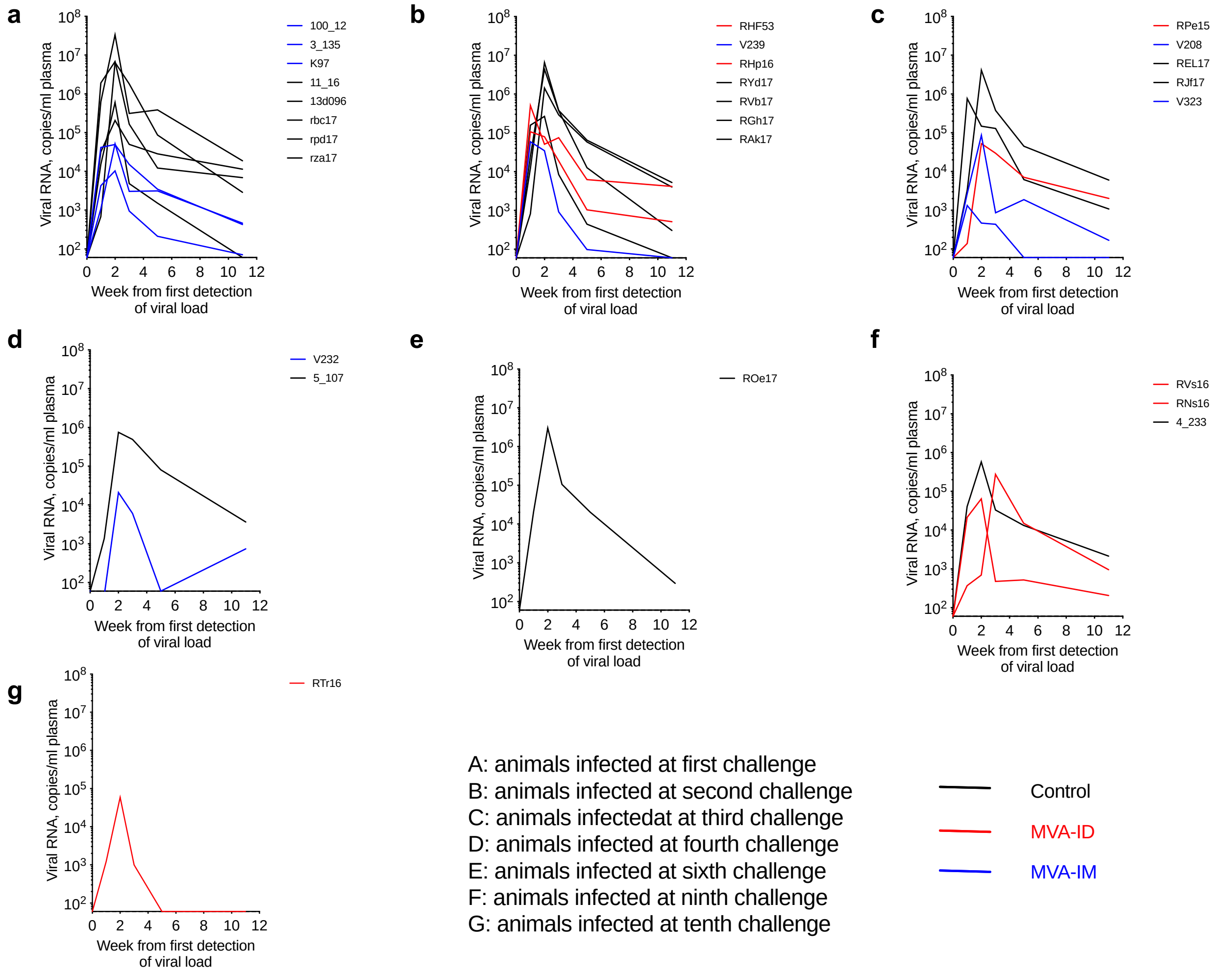

SFig. 3: Viral load kinetics of infected animals compared by challenge number they got infected. a-g) Viral load kinetics of infected animals from all the experimental groups presented according to their challenge number they got infected. Groups were color-coded; Control: black, MVA-ID: red, MVA-IM: blue. Individual animals were coded by their IDs. Source data are provided as a Source Data file.

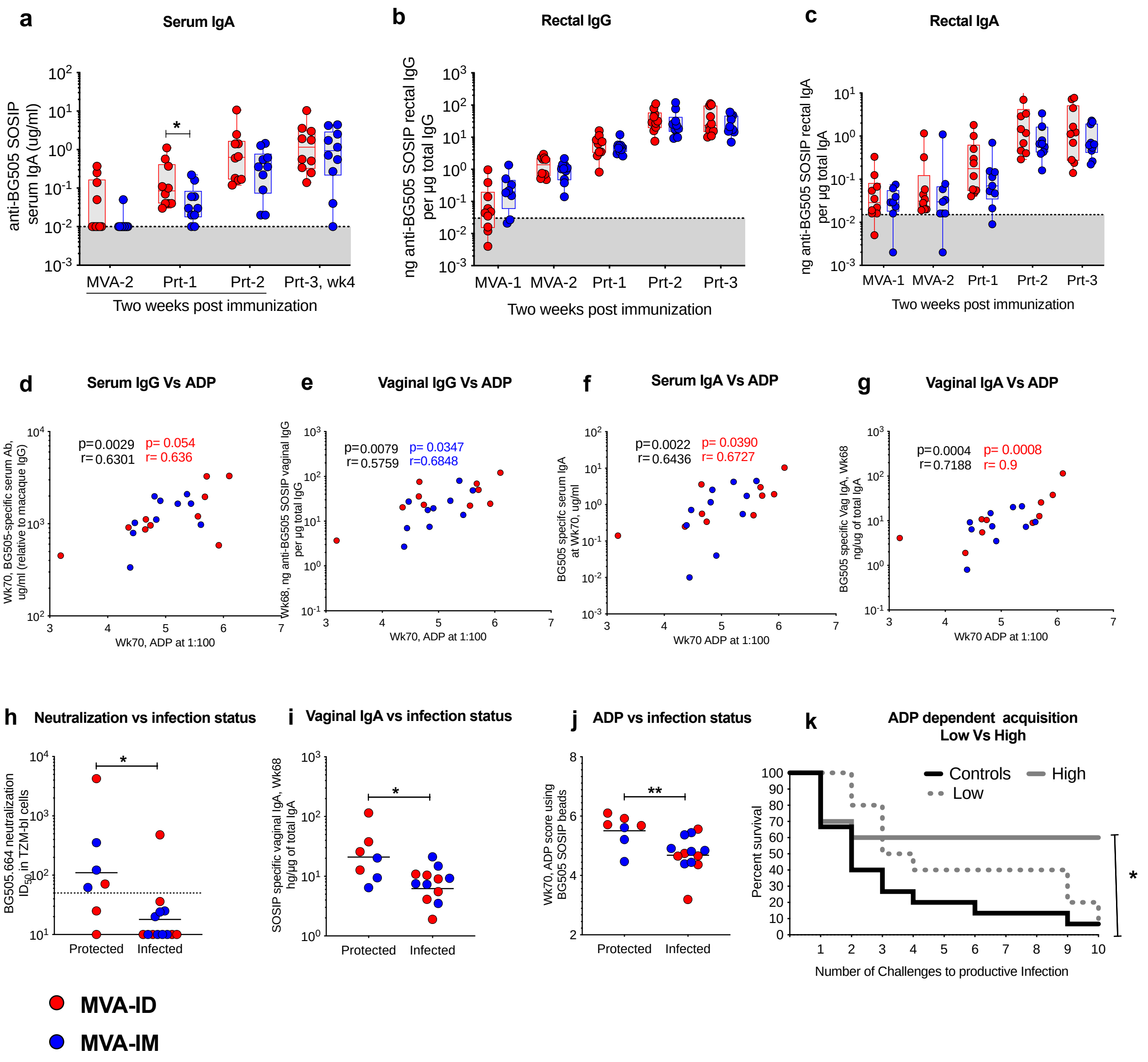

SFig. 4: Extended antibody data post vaccination

a) Serum anti-BG505_SOSIP binding IgA. Asterisks denote statistically significant differences at the given time point, measured by Mann-Whitney rank-sum (two tailed) test (*p=0.04) b-c) Anti BG505-SOSIP binding IgG and IgA in rectal secretions; n=10 in MVA-ID group at all time points; n=8 in MVA-IM at MVA-1 time point and n=10 at rest of the time points for measurement of IgG; n=8 in MVA-IM at MVA-1 and n=9 at MVA-2, Prt-1 time points and n=10 at rest of the time

points for measurement of IGA. Boxes in all the box plots extends from 25th percentile to 75th percentile of dataset and line inside the box denotes median. The whiskers outside the box descend to the dataset's minimum values and ascend to the maximum values. Spearman's correlation (two-sided) between serum ADP score and d) anti-BG505 SOSIP IgG, e) anti-BG505 SOSIP vaginal IgG. f) serum anti-BG505 SOSIP IgA, g) anti-BG505 vaginal IgA. In all the correlation plots, p and r values were color-coded to represent the vaccine group (red: MVA-ID, Blue: MVA-IM, Black: both groups combined). These values are not shown for the group that did not reach a statistically significant p value of 0.05. h-j) All vaccinated animals were clustered based on their protection status against h) serum neutralization titer (p=0.01), i) vaginal IgA (p=0.014) j) serum ADP activity (p=0.0063) (color-coded by groups); n=7 in protected group and n=13 in infected group. Asterisks denote statistically significant differences between the groups at the given time point, measured by Mann-Whitney rank-sum (two tailed) test. k) Kaplan-Meier curves showing the rate of infection in animals with low and high serum ADP scores on day of challenge (all vaccinated animals included and stratified as low and high, based on the median ADP score as cut-off) compared with control animals. Statistical significance was measured by log-rank test (*p<0.05). MVA-1: 1st MVA; MVA-2: 2nd MVA; Prt-1: 1st protein; Prt-2: 2nd protein; Prt-3: 3rd protein. Source data are provided as a Source Data file.

a

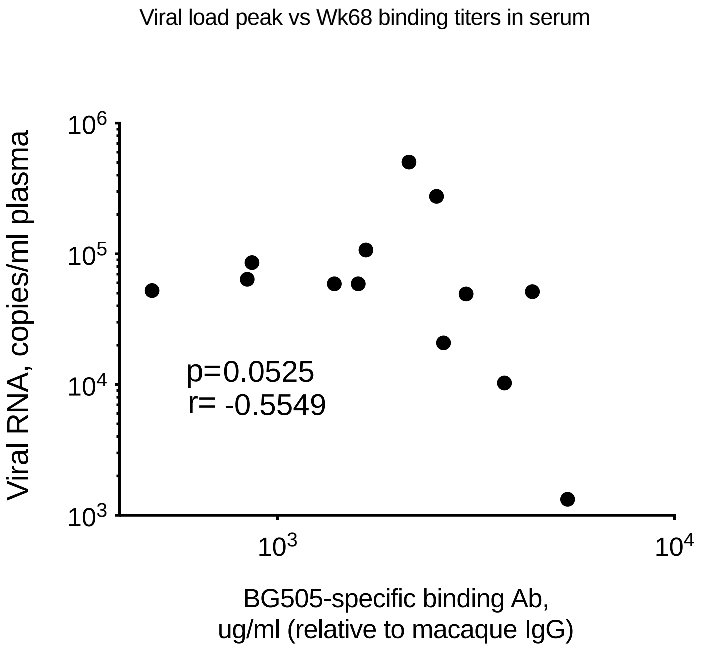

b

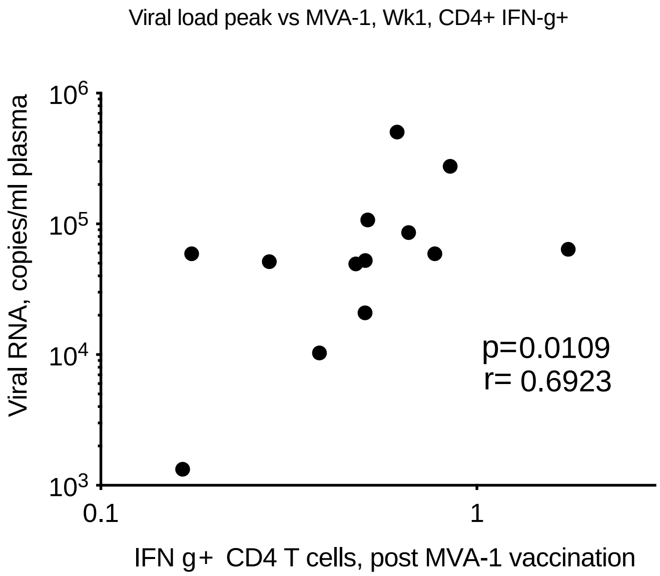

SFig. 5: Correlation of post-challenge peak viral load with pre-challenge vaccine induced antigen-specific immune response

Spearman correlations (two sided) between peak viral load and a) Wk68, BG505-specific binding antibody in the serum and b) MVA-1, Wk-1, IFNγ+ CD4 T cells in the blood. All infected animals from both the groups were included for the analysis. The p and r values represent two-tailed p values and Spearman's r. MVA-1: 1st MVA. Source data are provided as a Source Data file.

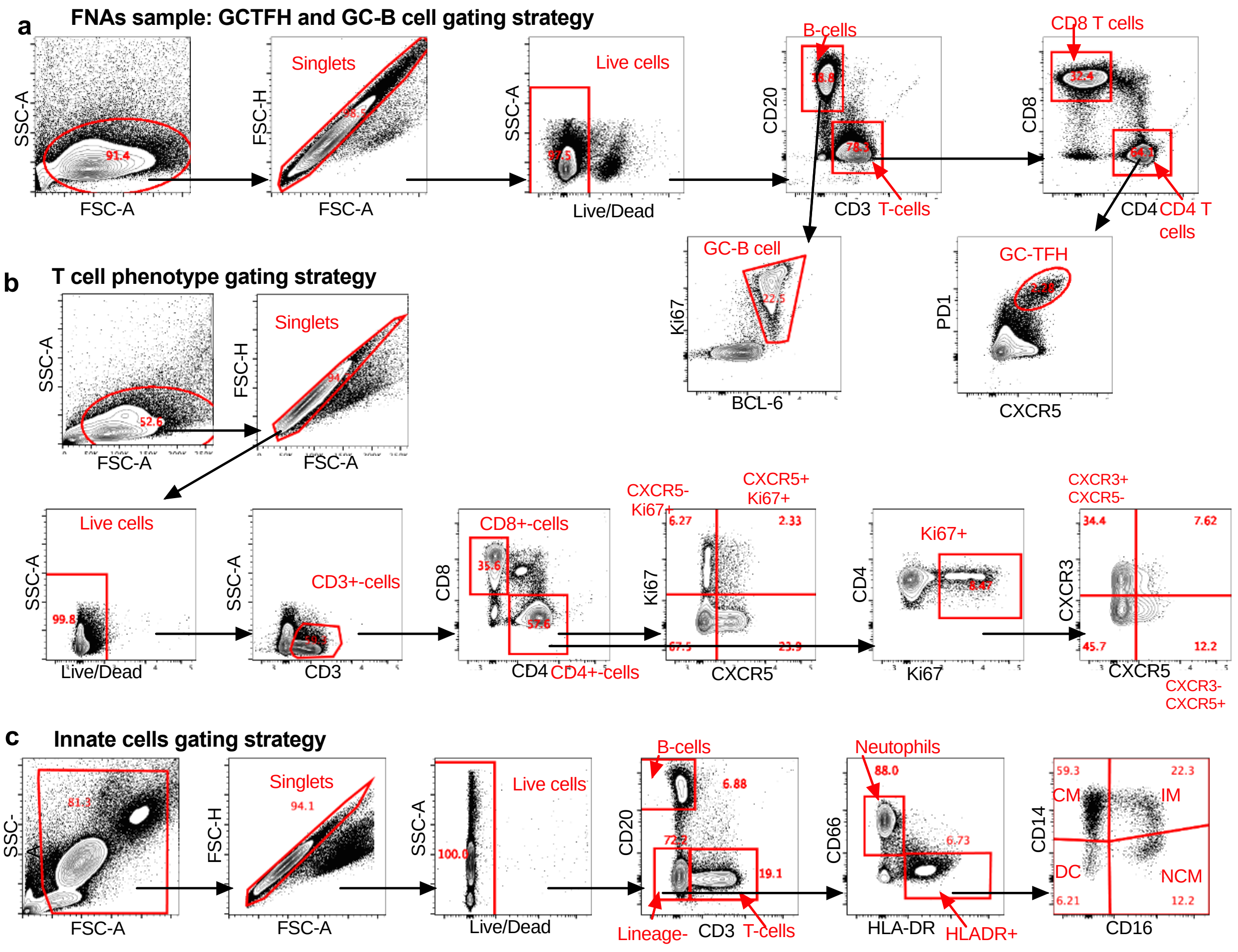

SFig. 6: Gating strategies used in flowcytometric analysis

Gating strategy used to identify a) GC-Tfh and GC-B cells from draining LN FNAs processed cells. b) CXCR5+/- Ki67+ cells and CXCR3+ CXCR5-, CXCR3-CXCR5+ cells from frozen PBMCs. c) Monocyte sub-sets and DC from whole blood. CM: classical monocytes; IM: intermediate monocytes; NCM: Non-classical monocytes; DC: Dendritic cells.

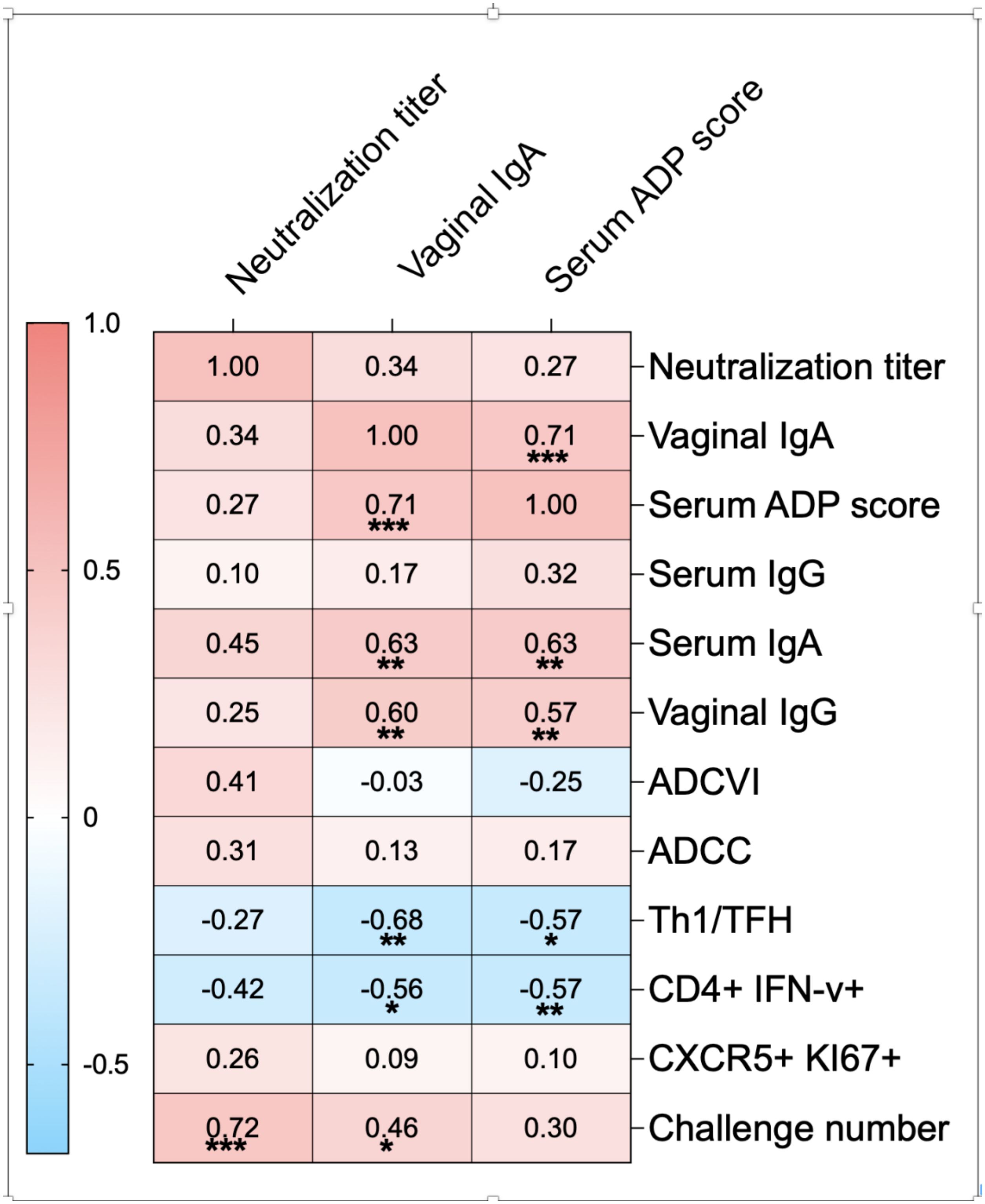

SFig. 7: Correlation matrix between protection correlates and other cellular parameters

Protection correlates serum neutralization titer (Wk70), vaginal IgA (Wk68), serum ADP score (Wk70) as variables on X-axis were plotted against protection correlates themselves and other parameters like serum IgG (Wk68), serum IgA (Wk70), Vaginal IgG (Wk68), ADCVI(Wk68), ADCC (Wk68), Th1/TFH , CD4+IFNγ+, CXCR5+ Ki67+ (all CD4 T cell parameters at MVA-1, Wk1), challenge number of infection on the Y axis. Positive correlation between the variables was denoted on a gradient of red color and negative correlation between the variables was denoted on a gradient of blue color. In all the correlations included in the matrix, r and p values were included in the respective matrix box. Asterisks denote statistical significance from a spearman correlation (two sided)(Neutralization titer Vs challenge number:***p=0.0003; Vaginal IgA Vs serum ADP:***p=0.0004, serum IgA:**p=0.003, vag IgG: **p=0.004, Th1/Tfh: **p=0.001, CD4+ IFNg+:*p=0.012, challenge number:*p=0.04; Serum ADP Vs Vag IgA:***p=0.0004, serum IgA: **p=0.003, vag IgG: **p=0.007, Th1/Tfh: *p=0.012, CD4+ IFNg+: **p=0.008). Source data are provided as a Source Data file.

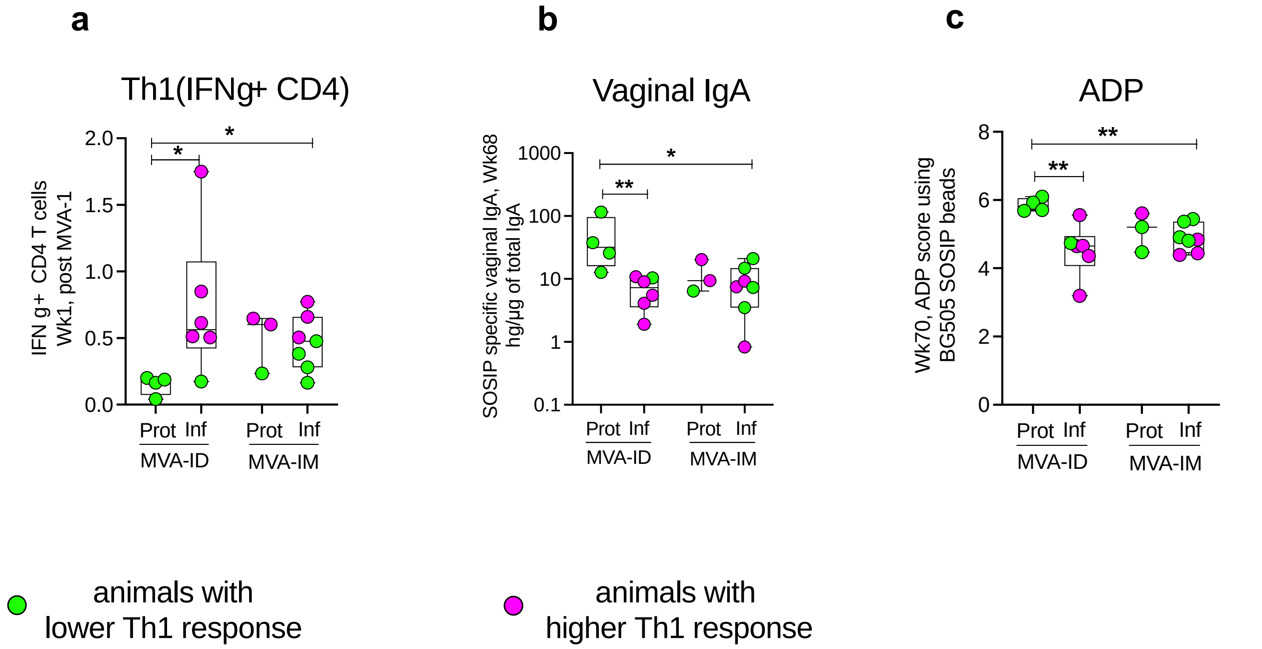

SFig. 8: Combination of lower Th1, higher vaginal IgA and serum ADP activity associates with protection in vaccinated animals.

a) IFN- γ+ CD4 T cells measured at Day7 post MVA-1. b) Vaginal IgA measured at Wk68. c) Serum ADP score measured at Wk70 in protected and infected animals within a group and comparing both vaccinated groups. Animals are color-coded with green: low IFN- γ+ CD4 T cells and pink: high IFN- γ+ CD4 T cells; n=4 in MVA-ID protected; n=6 in MVA-ID infected; n=3 in MVA-IM protected; n=7 in MVA-IM infected groups. Boxes in all the box plots extends from 25th percentile to 75th percentile of dataset and line inside the box denotes median. The whiskers outside the box descend to the dataset's minimum values and ascend to the maximum values. Asterisks denote statistical significance measured by Mann-Whitney rank-sum (two tailed) test (Th1: Protected MVA-ID Vs infected MVA-ID: *p=0.038, infected MVA-IM: *p=0.042; Vaginal IgA: Protected MVA-ID Vs infected MVA-ID: **p=0.009, infected MVA-IM: *p=0.024; ADP: Protected

MVA-ID Vs infected MVA-ID: ***p=0.009, infected MVA-IM: **p=0.006) . MVA-1: 1st MVA. Source data are provided as a Source Data file.

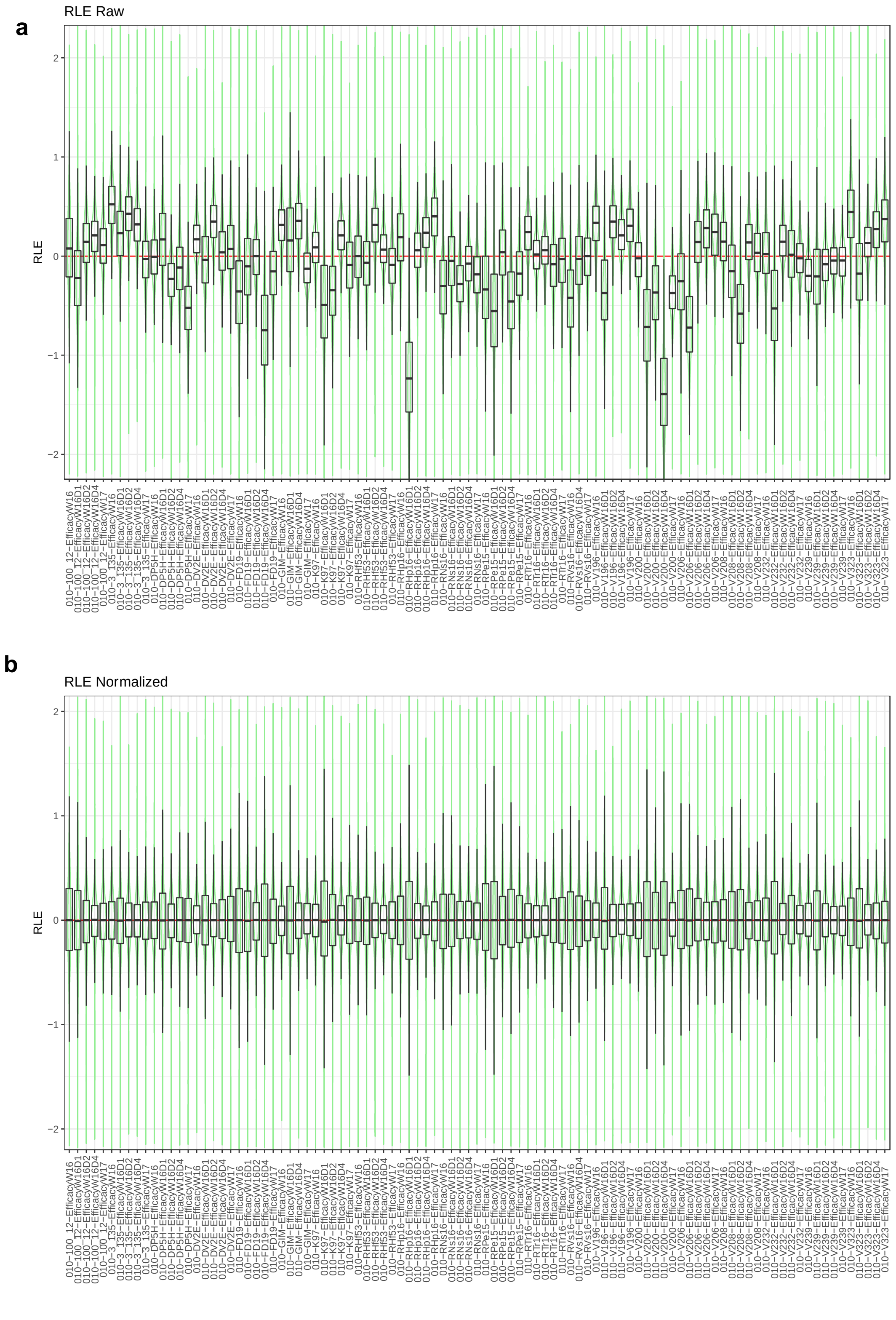

SFig. 9: RLE plots and normalization in RNA seq

a) Raw RLE plot before normalization and b) RLE plot after normalization, representing variation in expression of all genes measured in each sample. The line at 0 on Y axis shows the median expression of all genes in each sample; n=11495 genes per sample. Boxes in all the box plots extends from 25th percentile to 75th percentile of dataset and line inside the box denotes median. The extending whisker lines shows 1.5* IQR (inter-quartile range) beyond the box: less than the 25th percentile and greater than the 75th percentile. Green line behind the box plot is a violin plot that shows the full extent of the data minimum to maximum.

Group	Animal code	MHC-1 allele			Challenge Cohort	Weight at challenge	Age at challenge	Neutralization titers at the time of challenge	Number of challenges took for infection
		A*001	B*008	B*017					
MVA-ID	RVs16	-	-	-	1	6.29	4	10	9
	RTr16	-	+	-	1	5.49	4	479	10
	RPe15	-	-	-	1	9.02	6	10	3
	RHp16	-	-	-	1	7.69	4	10	2
	RNs16	-	-	-	1	6.99	4	36	9
	RHF53	-	-	-	1	6.64	13	10	2
	DP5H	-	-	-	2	8.43	11	4255	-
	GIM	+	-	-	2	8.03	12	71	-
	DV2E	-	-	-	2	8.82	10	25	-
	FD19	-	-	-	2	5.74	9	10	-
MVA-IM	V200	-	-	-	2	9.03	6	62	-
	V208	-	-	-	2	9.89	6	10	3
	V232	-	-	+	2	8.17	6	25	4
	V239	-	-	-	2	11.15	6	10	2
	100_12	-	-	-	2	5.61	6	20	1
	3_135				2	9.75	5	10	1
	V196	-	-	+	2	8.85	6	122	-
	V206	-	-	-	2	7.26	6	354	-
	V323	-	+	-	2	6.97	6	24	3
	K97	-	-	-	2	5.15	9	10	1
3M-053 only	11_16	-	-	-	1	9.8	7		1
	13D096	-	-	-	1	7.19	5		1
	4_233	-	-	-	1	9.57	14		9
	5_107	+	-	-	1	9.2	13		4
	RAk17	+	-	-	2	4.9	3		2
	RBc17	+	-	-	1	5.72	3		1
	REl17	+	-	-	2	6.09	3		3
	RGh17	-	-	-	1	6.46	3		2
	RJf17	+	-	-	1	6.13	3		3
	ROe17	-	-	-	2	6.33	3		6
	RPd17	-	-	-	2	5.9	3		1
	RVb17	-	-	-	1	5.82	3		2
	RYd17	-	-	-	1	5.55	3		2
	RZa17	-	-	-	2	5.22	3		1
	RZs9	-	-	-	1	9.23	15		-

The first cohort of animals were challenged from September 2018 through February 2019. The second cohort of animals were challenged from March 2019 through July 2019. Six of the 20 vaccinated animals from the current study were part of cohort 1 and the remaining 14 were part of cohort 2. We included 10 control animals with cohort 1 and 5 controls with cohort 2, and these were added few months before the challenge.

STable. 1: Animal assignment and details

Supplementary information:

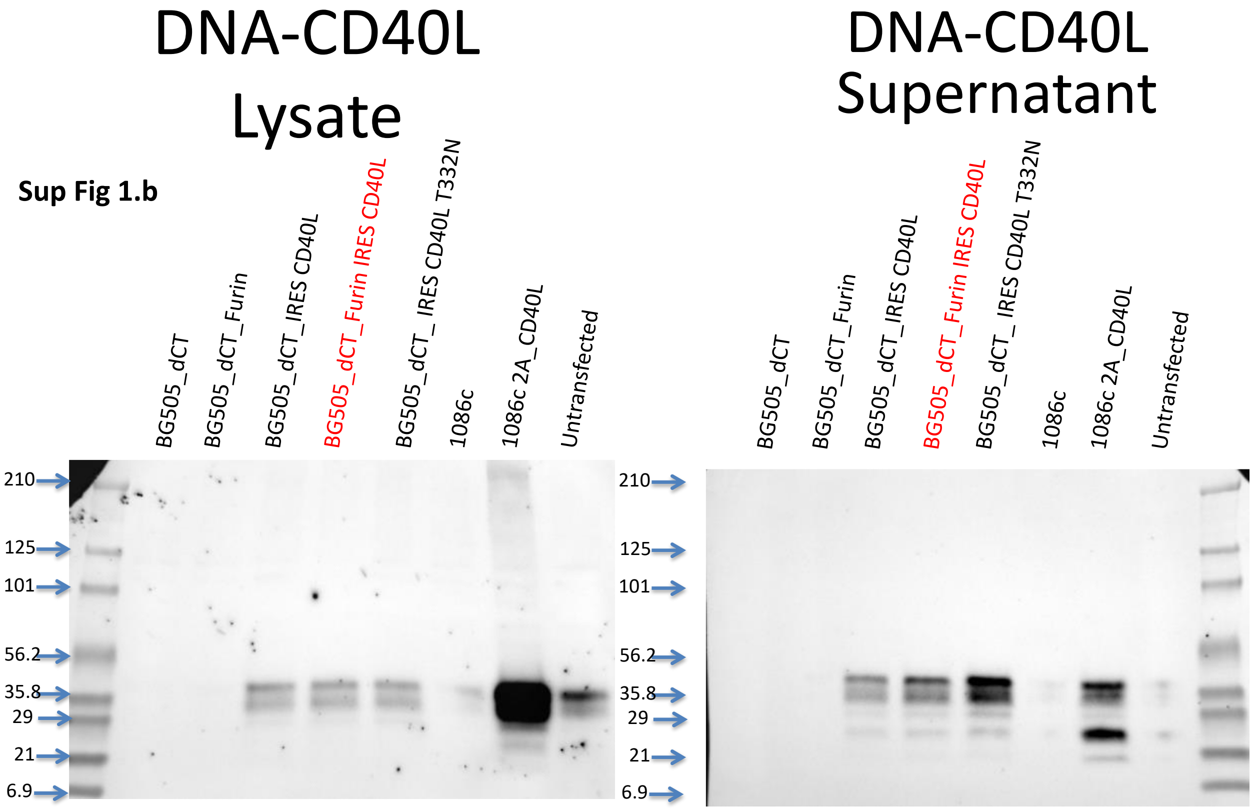

Sup Fig 1.b

DNA-ENV Lysate

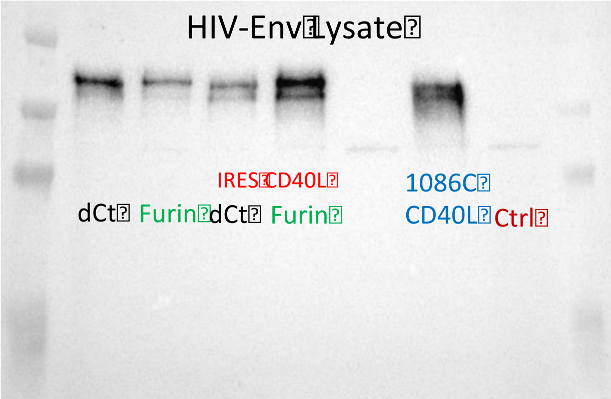

DNA-ENV Supernatant

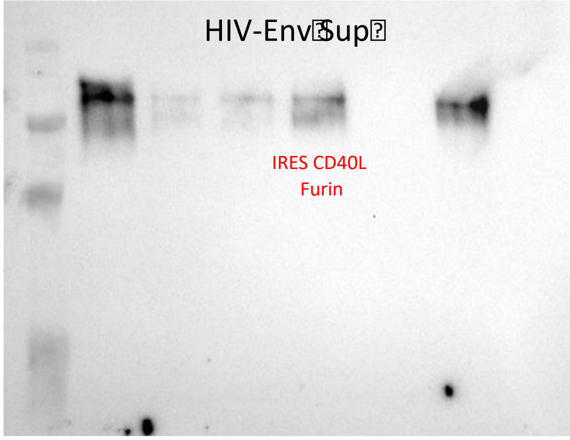

Sup Fig 1.b

DNA-Gag Lysate, Supernaant

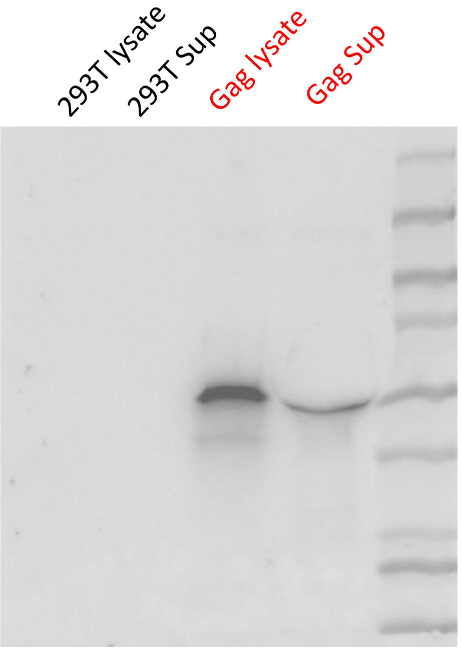

Sup Fig 2.b

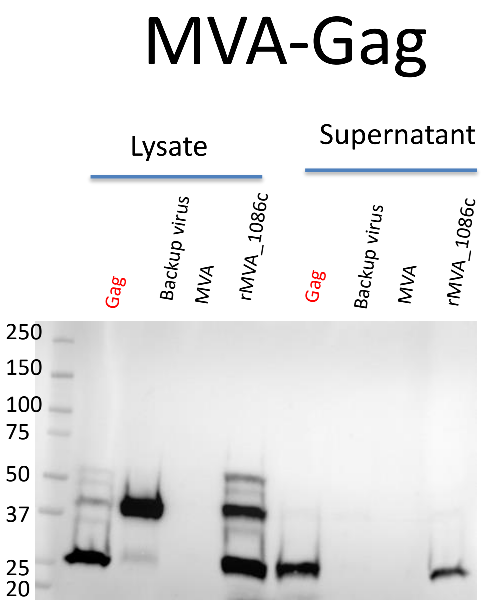
MVA-Gag
Lysate
Supernatant
Gag
Backup virus
MVA
rMVA_1086c
Gag
Backup virus
MVA
rMVA_1086c
250
150
100
75
50
37
25
20

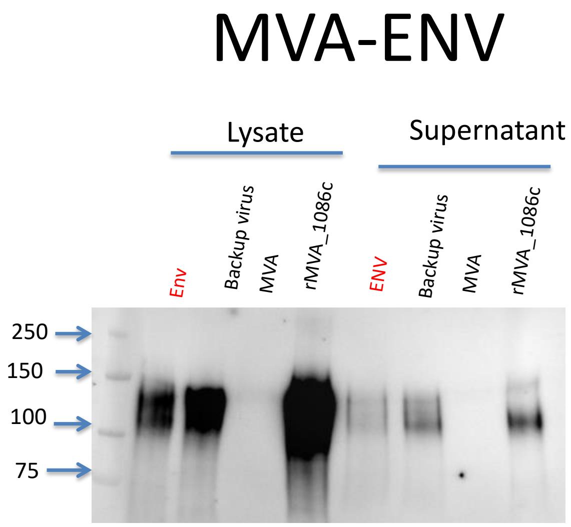
MVA-ENV
Lysate
Supernatant
Env
Backup virus
MVA
rMVA_1086c
ENV
Backup virus
MVA
rMVA_1086c
250
150
100
75
