# Supplementary material for: Intradermal but not intramuscular modified vaccinia Ankara immunizations protect against intravaginal tier2 simian-human immunodeficiency virus challenges in female macaques
Source: Nat Commun. 2023 Aug 8;14:4789. doi: 10.1038/s41467-023-40430-7 (PMC10409804; doi:10.1038/s41467-023-40430-7)
Supplement: Supplementary file 3 — Reporting Summary [file 41467_2023_40430_MOESM3_ESM.pdf]

nature portfolio

Corresponding author(s): Rama Rao Amara

Last updated by author(s): Jun 30, 2022

Reporting Summary

Nature Portfolio wishes to improve the reproducibility of the work that we publish. This form provides structure for consistency and transparency in reporting. For further information on Nature Portfolio policies, see our Editorial Policies and the Editorial Policy Checklist.

Statistics

For all statistical analyses, confirm that the following items are present in the figure legend, table legend, main text, or Methods section.

n/a	Confirmed	
☐	☒	The exact sample size (*n*) for each experimental group/condition, given as a discrete number and unit of measurement
☐	☒	A statement on whether measurements were taken from distinct samples or whether the same sample was measured repeatedly
☐	☒	The statistical test(s) used AND whether they are one- or two-sided<br>*Only common tests should be described solely by name; describe more complex techniques in the Methods section.*
☐	☒	A description of all covariates tested
☐	☒	A description of any assumptions or corrections, such as tests of normality and adjustment for multiple comparisons
☐	☒	A full description of the statistical parameters including central tendency (e.g. means) or other basic estimates (e.g. regression coefficient) AND variation (e.g. standard deviation) or associated estimates of uncertainty (e.g. confidence intervals)
☐	☒	For null hypothesis testing, the test statistic (e.g. *F*, *t*, *r*) with confidence intervals, effect sizes, degrees of freedom and *P* value noted<br>*Give P values as exact values whenever suitable.*
☒	☐	For Bayesian analysis, information on the choice of priors and Markov chain Monte Carlo settings
☒	☐	For hierarchical and complex designs, identification of the appropriate level for tests and full reporting of outcomes
☐	☒	Estimates of effect sizes (e.g. Cohen's *d*, Pearson's *r*), indicating how they were calculated

Our web collection on statistics for biologists contains articles on many of the points above.

Software and code

Policy information about availability of computer code

Data collection	BD F ACS DIVA Software v8.0 .l,
Data analysis	networkanalyst.ca 3.0 was used for pathway enrichment analysis in RNA seq experiment.DESeq2 version 1.22.1 R package was used to produce normalized read counts in RNA seq experiment. Graphpad prism versions 8 and 9 were used for statistical analysis, STAR version 2.2.2b was used to map RNA reads.

For manuscripts utilizing custom algorithms or software that are central to the research but not yet described in published literature, software must be made available to editors and reviewers. We strongly encourage code deposition in a community repository (e.g. GitHub). See the Nature Portfolio guidelines for submitting code & software for further information.

Data

Policy information about availability of data

All manuscripts must include a data availability statement. This statement should provide the following information, where applicable:

- Accession codes, unique identifiers, or web links for publicly available datasets
- A description of any restrictions on data availability
- For clinical datasets or third party data, please ensure that the statement adheres to our policy

Source data are provided with this paper. RNA-seq data used in this study is available in GEO repository under accession code GSE219118 (https://www.ncbi.nlm.nih.gov/geo/query/acc.cgi?acc=GSE219118). Indian rhesus macaque genomic reference 53 available at: https:// www.unmc.edu/

rhesusgenechip/index.htm. clade D Env sequence with Gen Bank accession:KU958484.1 is available at (https:// www.ncbi.nlm.nih.gov/nuccore/KU958484.1/).

Research involving human participants, their data, or biological material

Policy information about studies with human participants or human data. See also policy information about sex, gender (identity/presentation), and sexual orientation and race, ethnicity and racism.

Reporting on sex and gender	N/A
Reporting on race, ethnicity, or other socially relevant groupings	N/A
Population characteristics	N/A
Recruitment	N/A
Ethics oversight	N/A

Note that full information on the approval of the study protocol must also be provided in the manuscript.

Field-specific reporting

Please select the one below that is the best fit for your research. If you are not sure, read the appropriate sections before making your selection.

[x] Life sciences   [ ] Behavioural & social sciences   [ ] Ecological, evolutionary & environmental sciences

For a reference copy of the document with all sections, see nature.com/documents/nr-reporting-summary-flat.pdf

Life sciences study design

All studies must disclose on these points even when the disclosure is negative.

Sample size	We chose 10 female rhesus macaques in the vaccinated group. We choose this number based on the power calculations as described by Hudgens et al (The Journal of Infectious Diseases 2009; 200:609–13) and assuming an infection rate of ~0.3 in the control arm. These analyses revealed that we will achieve 80% or higher power if the risk ratio (ratio of rate of infection in vaccinated over rate of infection in controls) is 0.37 or lower.
Data exclusions	No data was excluded from the study.
Replication	Characterizations of immunogens, neutralization data, binding antibody data and all in-vitro experiments are reproducible. The vaccination and viral challenge experiment is not repeated as it is two year long study and it is expensive. In-vitro experiments were repeated twice and we have noticed similar observations.
Randomization	Animals were randomly distributed between the groups based on age and weight. Samples were not randomized when used for analyses. Samples from all vaccinated and control animals (where applicable) were analyzed at the same time to control for any possible experimental error.
Blinding	Veterinarians that performed NHP study were blinded to study design. Many analyses such as neutralizing antibody, viral load measurements, ADCVI, ADCC and ADP were done on a blinded fashion. T cell assays were not performed on a blinded fashion since these were performed on fresh cells as samples were being collected. This was not an issue since each sampling group contained animals from different experimental groups.

Reporting for specific materials, systems and methods

We require information from authors about some types of materials, experimental systems and methods used in many studies. Here, indicate whether each material, system or method listed is relevant to your study. If you are not sure if a list item applies to your research, read the appropriate section before selecting a response.

Materials & experimental systems

n/a	Involved in the study
☐	☒ Antibodies
☐	☒ Eukaryotic cell lines
☒	☐ Palaeontology and archaeology
☐	☒ Animals and other organisms
☒	☐ Clinical data
☒	☐ Dual use research of concern
☒	☐ Plants

Methods

n/a	Involved in the study
☒	☐ ChIP-seq
☐	☒ Flow cytometry
☒	☐ MRI-based neuroimaging

Antibodies

Antibodies used

BV-650 CD20: Biolegend, 302335, 2H7, 4 ml in 100 ml
BV-510 CD14: Biolegend 301842, M5E2, 5 ml in 100 ml
PERCP HLADR: BD 347364, L243, 10 ml in 100 ml
APC CD66: Miltenyi 130-118-539, TET2, 3 ml in 100 ml
BV-711 CD16: BD 563127, 3G8, 5 ml in 100 ml
BV-605 CD86: Biolegend 305430, IT2.2, 4 ml in 100 ml
PERCP CD3: BD 552851, SP34-2, 5 ml in 100 ml
BV-650 CD4: Biolegend 317436, OKT4, 0.1 ml in 100 ml
PEcy7 CD20: Biolegend 302312, 2H7, 4 ml in 100 ml
BV-421 PD-1: Biolegend 329920, EH12.2H7, 3 ml in 100 ml
PE CXCR5: Invitrogen 12-9185-42, MU5UBEE, 4 ml in 100 ml
APC CD8: BD 340584, SK1, 3 ml in 100 ml
A700 CD3: BD 557917, SP34-2, 3 ml in 100 ml
BV-711 CD8: Biolegend 344734, SK1, 5 ml in 100 ml
BV-605 CXCR3: Biolegend 353728, G025H7, 1 ml in 100 ml
FITC BCL6: Biolegend 358514, 7D1, 5 ml in 100 ml
A700 Ki67: BD 561277, B56, 5 ml in 100 ml
PEcY7 Ki67: BD 561283, B56, 5 ml in 100 ml
Anti-CD28: BD 555725, CD28.2, 0.2 ml in 100 ml
Anti-CD49d: BD 555501, 9F10, 0.2 ml in 100 ml
BV-510 CD8: BD 563919, SK1, 0.02 ml in 100 ml
A700 IFN-γ: BD 557995, B27, 3 ml in 100 ml
PE-CF594 TNF-α: BD 562784, MAB11, 2 ml in 100 ml
BV-421 MIP1β: BD 562900, D21-1351, 3 ml in 100 ml

Validation

All antibodies and reagents used in our study were validated by vendors, sources and in our experimental positive controls. All antibodies used are commercially validated on the manufacturer's website as flow cytometry reagents raised against macaque antigens or validated to cross-react with macaque antigens according to the manufacturer's website.

Eukaryotic cell lines

Policy information about cell lines and Sex and Gender in Research

Cell line source(s)

Dfl cells: Source-ATCC
HEK-293T: Source-ATCC
CHO cells: Source-ATCC
TZM-bl: Source-ATCC
CCRS+ CEM-NKr cells: obtained from David Evans
Rhesus CD16 expressing KHYGl NK effector cells: obtained from Prof. David Evans
THP-1 monocytic cells: Source-ATCC

Authentication

The cell lines were authenticated using short-tandom repeat determination through Cell Authentication services of ATCC. Reports can be obtained on request. CCRS+ CEM-NKr cells, Rhesus CD16 expressing KHYGlNK effector cells were obtained from Prof .. David Evans and also authenticated by him.

Mycoplasma contamination

Negative

Commonly misidentified lines
(See ICLAC register)

None

Animals and other research organisms

Policy information about studies involving animals; ARRIVE guidelines recommended for reporting animal research, and Sex and Gender in Research

Laboratory animals	Rhesus macaque, Age 3 years to 15 years
Wild animals	Study did not involve wild animals
Reporting on sex	As our study involved intra-vaginal route of virus challenge, we used only female rhesus macaques to evaluate protection efficacy of our vaccine against infection.
Field-collected samples	The study did not involve samples collected form the field.
Ethics oversight	Institutional Animal Care and Use Committee (IACUC) of Emory University approved all the procedures involving animals

Note that full information on the approval of the study protocol must also be provided in the manuscript.

Flow Cytometry

Plots

Confirm that:

- [x] The axis labels state the marker and fluorochrome used (e.g. CD4-FITC).
- [x] The axis scales are clearly visible. Include numbers along axes only for bottom left plot of group (a 'group' is an analysis of identical markers).
- [x] All plots are contour plots with outliers or pseudocolor plots.
- [x] A numerical value for number of cells or percentage (with statistics) is provided.

Methodology

Sample preparation	Draining lymph nodes were identified by veterinarians and cells were collected using 22-gauge needle attached to 3m syringe by passing 4 times into the lymph node. These samples were suspended in RPMI medium containing 10% FBS, 1X peniaillin/streptomycin. Samples were centrifuged and ACK (ammonium-chloride-potassium) lysis buffer was added if the sample had red blood cells. Frozen PBMCs were carefully thawed, washed thrice with warm RPMI medium containing 10% FBS and 1X peniciin /streptomycin, and stained, Blood samples and frozen PBMCS were surface stained with respective innate cell marker antibodies and T-cell phenotype antibodies. PBMCs were stimulated with overlapping 1ug/ml, 8G505 Env peptide pools or lue/mL, SIVmac239 Gag peptide pool(Cat: 12364, HIV reagent program) in the presence of co-stimulants lug/mL, anti-CD28 and 1ug/ml, anti-CD49d (BD pharmingen, San Diego, CA). BG505 Env poal (NIH ARP cat 13123) consistec of 213 overlapping 15mer peptides (overlapped by 11 residues). Env-1 pool consisted of first 106 peptides and rest was in Env-2 pool. After 2 hrs of stimulation at 37"C in 5% C02, 0.5ug/ml of Brefeldin-A (BD pharmingen] and 0.5ug/mt of Golgistop (BD pharmingen) were added per each well. After 4hr of further incubation, cells were stored at 4'C avernight. Next day morning, cells were washed in FACS wash buffer (PBS with 2%FBS and 0.05% sodium azide)] and surface stained with PERCP anti-human CD3, BV-650 anti - human CD4, BV510 anti-human CD8 and live/Dead stain for 30 min at room temperature. Cells were washed and then permeabilized with cytofix/cytoperm (BD biosciences) for 20 min at 4'C and washed with perm wash buffer (BD biosciences). These cells were stained intracellularly with A700 anti-human IFN-¥; PE-CF594 anti-human TNF-a and BV-421 anti-human MIP1f for 30 min at 4"C. Cells were washed with perm wash buffer and finally suspended in FACS wash buffer
Instrument	These cells were acquired using LSRII instrument (BD immunocytometry systems, san jose, CA)
Software	analyzed using flowjo software (Treestar, Ashland, OR)
Cell population abundance	no cell sorting was used in this study
Gating strategy	GCTFH and GC-Bcell gating strategy:<br>Cells were plotted against SSC-A and FSC-A and cells with lower Side scatter were gated, these cells were plotted against FSC-H and FSC-A and cells placed diagonally were gated as singlets, these cells were plotted against live/dead dye and cells from the negative population of live dead stain were gated as live cells. these cells were plotted against CD3 and CD20 and their + populations are gated as CD3 T-cells and B-cells respectively, CD3 were plotted against CD4 and CD8 markers and their + populations are gated as CD4 T-cells and CD8 T-cells respectively, CD4 cells were plotted against CXCR5 and PD1 and ++ population are gated as GC-TFH, CD20 cells were plotted against BCL-6 and Ki67 and ++ population are gated as GC-B cells.<br><br>T cell phenotype gating strategy:<br>Cells were plotted against SSC-A and FSC-A and cells with lower Side scatter were gated, these cells were plotted against FSC-H and FSC-A and cells placed diagonally were gated as singlets, these cells were plotted against live/dead dye and cells from the negative population of live dead stain were gated as live cells. These cells were plotted against SSC-A and CD3, and CD3 were plotted against CD4 and CD8 markers and their + populations are gated as CD4 T-cells and CD8--cells respectively, CD4

cells were plotted against CXCR5 and Ki67 with a quadrant gate. CD4 cells were also plotted against Ki67 alone and cells from the + gate were plotted against CXCR5 and CXCR3 with a quadarant gate.

Innate cell gating strategy:
Cells were plotted against SSC-A and FSC-A and cells except double negative entire population was gated, these cells were plotted against FSC-H and FSC-A and cells placed diagonally were gated as singlets, these cells were plotted against live/dead dye and cells from the negative population of live dead stain were gated as live cells. these cells were plotted against CD3 and CD20 and their + populations are gated as CD3 T-cells and B-cells respectively and double negative cells are gated as lineage -ve. These lineage -ve cells were plotted against HLADR and CD66, and their + population are gated as HLADR+ and neutrophils respectively. HLADR+ cells were plotted against CD16 and CD14 with quadrant gates and Double negative cells were gated as DCs, double + are gated as IM cells and their + populations are gated as NCM and CM cells respectively.

☒ Tick this box to confirm that a figure exemplifying the gating strategy is provided in the Supplementary Information.
